# Vertical distance from navel as a risk factor for bowel obstruction associated with feeding jejunostomy after esophagectomy: a retrospective cohort study

**DOI:** 10.1186/s12876-020-01506-6

**Published:** 2020-10-27

**Authors:** Teppei Kamada, Hironori Ohdaira, Hideyuki Takeuchi, Junji Takahashi, Rui Marukuchi, Eisaku Ito, Norihiko Suzuki, Satoshi Narihiro, Sojun Hoshimoto, Masashi Yoshida, Mitsuyoshi Urashima, Yutaka Suzuki

**Affiliations:** 1 Department of Surgery, International University of Health and Welfare Hospital, Nasushiobara City, Tochigi 537-3, Iguchi329-2763 Japan; 2 Division of Molecular Epidemiology, Jikei University School of Medicine, Tokyo, Japan

**Keywords:** Bowel obstruction, Feeding jejunostomy, Esophageal cancer, Esophagectomy, Complications

## Abstract

**Background:**

Placement of feeding jejunostomy (PFJ) during esophagectomy is an effective method to maintain adequate nutrition, but is associated with serious complications such as bowel obstruction and jejunal torsion. The purpose of the current study was to analyze the incidence, clinical features, and risk factors of bowel obstruction associated with feeding jejunostomy (BOFJ) after PFJ.

**Methods:**

This was a retrospective cohort study of 70 patients who underwent esophagectomy with three-field lymph node dissection for esophageal cancer and treated with PFJ between March 2013 and December 2019 in our hospital. Abdominal dissection was performed under hand-assisted laparoscopic surgery (HALS) from March 2013 to March 2015, and was changed to complete laparoscopic surgery in April 2015. We compared patients with and without BOFJ, and the incidence of BOFJ was evaluated. The primary endpoint was incidence of BOFJ after PFJ.

**Results:**

Six patients (8.5%) were diagnosed with BOFJ, all of whom were symptomatic and in the HALS group. In addition, 3 cases displayed histories of recurrent BOFJ (3, 3, and 5 times). Laparotomy was performed in all cases. Subgroup analysis of the HALS group showed a significant difference only in straight-line distance between the jejunostomy and navel as a significant pre- and perioperative factor (117 mm [101–130 mm] vs. 89 mm [51–150 mm], *p* < 0.001). Furthermore, dividing straight-line distance between the jejunostomy and navel into VD and HD, only VD differed significantly (107 mm [93–120 mm] vs. 79 mm [28–135 mm], *p* = 0.010), not HD (48 mm [40–59 mm] vs. 46 mm [22–60 mm], *p* = 0.199).

**Conclusions:**

VD between the jejunostomy and navel was associated with BOFJ after PFJ with HALS esophagectomy. PFJ < 9 cm above the navel during HALS esophagectomy might effectively prevent BOFJ.

## Background

Esophagectomy for esophageal cancer is a highly invasive procedure with rates of postoperative complications (e.g., cardiovascular events, respiratory events, or anastomotic leakage) and mortality higher than those of other gastroenterological surgery [1–3]. Postoperatively, patients often experience loss of appetite and insufficient oral intake due to the invasiveness of the procedure and the anatomical changes caused by esophagectomy, the process of disease progression, and side effects from chemotherapy [4, 5]. This can then lead to weight loss. Placement of feeding jejunostomy (PFJ) during esophagectomy is recommended to maintain adequate nutrition in patients [6–8], but PFJ is associated with a 2–15% incidence of serious complications, such as bowel obstruction or jejunal torsion [9–12]. However, few reports have described the risk factors of bowel obstruction associated with feeding jejunostomy (BOFJ).

In our institution, abdominal dissection was performed under hand-assisted laparoscopic surgery (HALS) with a 7-cm midline incision positioned just inferior to the xiphoid process from March 2013 to March 2015. This was changed to complete laparoscopic surgery with a 5-cm midline incision at the navel for the purpose of duodenal mobilization during gastric tube reconstruction from April 2015. The proximity of the PFJ to the navel thus changed according to the position of this small incision. We hypothesized that PFJ in the epigastrium would represent a risk factor for BOFJ, since BOFJ cases were encountered more frequently up to March 2015.

The aim of the current study was therefore to explore the association between vertical distance from the navel and frequency of BOFJ.

## Methods

### Study design

This was a retrospective cohort study of 70 patients who underwent esophagectomy with three-field lymph node dissection for esophageal cancer and were treated with PFJ at the International University of Health and Welfare Hospital (Nasushiobara, Tochigi prefecture, Japan), between March 2013 and December 2019. The requirement for informed consent was waived because data were anonymized and retrospective. All data were subject to strict privacy policies, and all patients or their family members were given the chance to opt out. This study was approved by the institutional review board at the International University of Health and Welfare Hospital (Approval No. 13-B-389).

### Patients

Seventy patients who underwent esophagectomy with three-field lymph node dissection for esophageal cancer with PFJ were enrolled. Patients who were treated using lower esophagectomy via an abdominal approach (n = 2), patients who underwent esophageal bypass (n = 1), and patients who underwent gastrostomy only due to unresectable tumors (n = 1) were excluded. Surgical indications for esophageal cancer were based on the esophageal cancer practice guidelines 2017 edited by the Japan Esophageal Society [13].

Clinical staging was conducted according to the 11th edition of the Japan Esophageal Society’s Japanese Classification of Esophageal Cancer [14].

The surgical procedure comprised esophagectomy and lymphadenectomy with PFJ in all cases. The indication for PFJ was esophagectomy for all cases in this cohort.

Abdominal dissection was performed under HALS with a 7-cm midline incision positioned just inferior to the xiphoid process from March 2013 to March 2015, and under complete laparoscopic surgery with a 5-cm midline incision at the navel since April 2015.

Surgeries for all Japanese adult patients with histologically proven, surgically resectable tumor (cT1-3 N0-3 M0) were performed after assessing the ability of the patient to tolerate surgery. Neoadjuvant chemotherapy plus surgery was performed for clinical Stage II or III, except for cases with severe stenosis. Postoperative adjuvant chemotherapy was performed for postoperative Stage II or III disease. Neoadjuvant chemotherapy comprised two cycles of 5-fluorouracil and cisplatin. Tegafur/gimeracil/oteracil (S-1) was administered as postoperative adjuvant chemotherapy.

All operations were performed by an experienced single surgeon who was a licensed attending doctor for laparoscopic surgery. All patients in this study underwent the following standard operations. Thoracoscopic esophagectomy with mediastinal lymph node dissection was performed with the patient in a prone position. After thoracic procedure, patients were placed in a supine position, and neck dissection, gastric mobilization with abdominal dissection, and gastric tube or ileocolic reconstruction were performed. Finally, PFJ was performed.

### Vertical distance from navel to jejunostomy

**Fig. 1:**
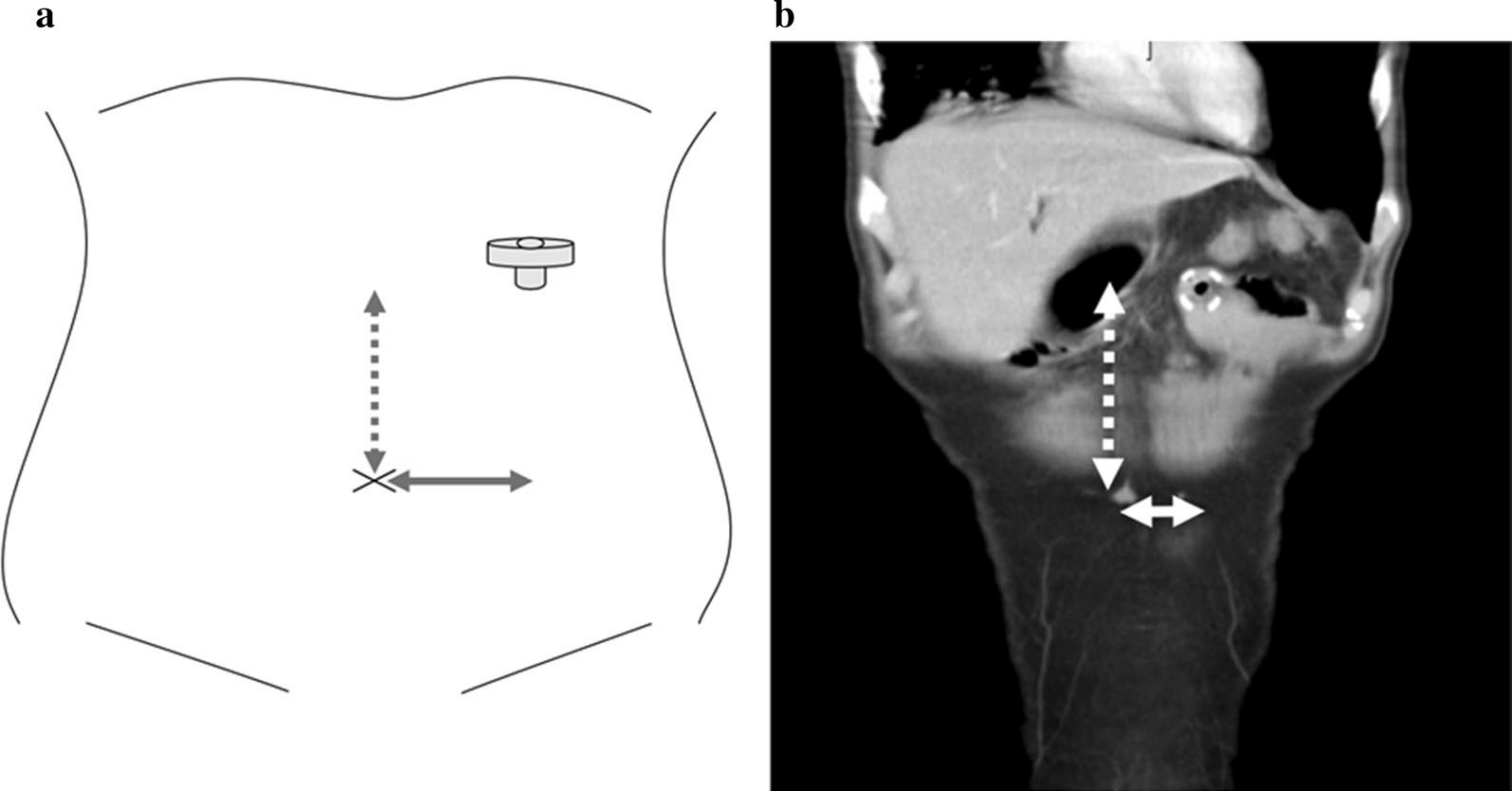
Measurement of straight-line distance (VD and HD) between the jejunostomy and navel performed from computed tomography (CT). **a** VD (dotted line arrow); **b** HD (solid line arrow)

Measuring the straight-line distance (as vertical distance [VD] and horizontal distance [HD]) between the jejunostomy and navel was performed from computed tomography (CT) taken within 1 month after primary operation (Fig. 1a, b).

In our institution, CT within 1 month after esophagectomy is routinely performed for the purpose of comparison with future follow-up CT and to rule out postoperative pneumonia and intraabdominal abscess [13].

The definition of the navel was the indented point of the midline incision.

### Surgical technique of PFJ

PFJ was performed under small laparotomy.

We applied button jejunostomy [15]. After esophagectomy, the ligament of Treitz was identified. The appropriate size of Ideal Button (IB) (Olympus Medical Systems Co, Tokyo, Japan) was determined depending on the thickness of the abdominal wall. At 20 cm distal from the ligament of Treitz, the IB was inserted through a 1-cm jejunal incision and a purse-string suture with 3-0 absorbable braid was performed to fix the IB. A 14-Fr, nasogastric tube (NG tube) was inserted 20 cm to the anal side of the jejunostomy through the IB to prevent jejunostomy leakage. The IB was then pulled out toward the extra-abdominal wall with a Kelly clamp. Two or three fixed abdominal wall-jejunum sutures with 3-0 absorbable braid were performed around the IB to achieve longitudinal fixation. Enteral nutrition through the jejunostomy was initiated on postoperative day 1. The 14-Fr NG tube was removed on postoperative day 7.

These procedures are consistent in all patients.

### Definition of BOFJ

**Fig. 2:**
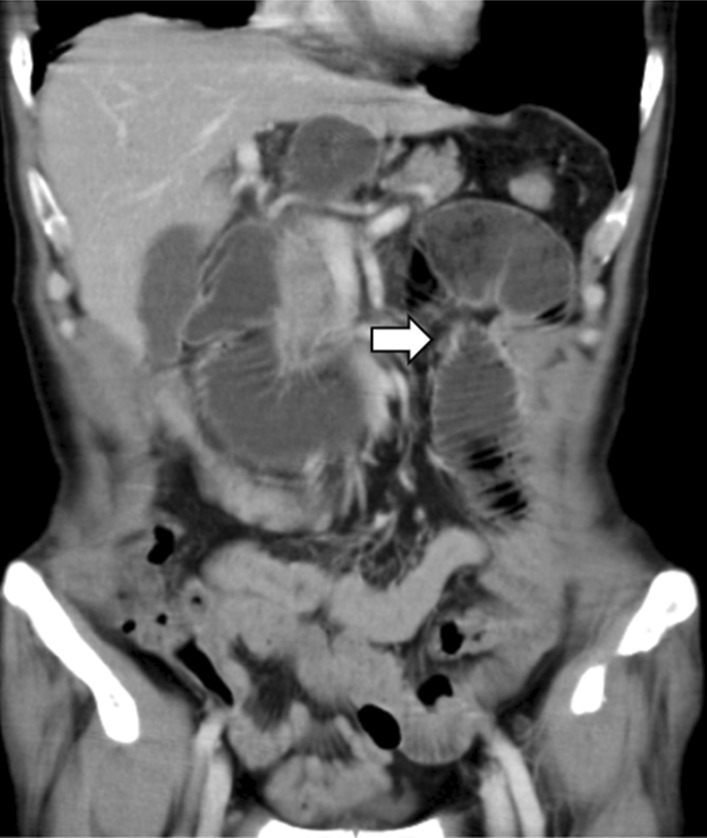
Contrast-enhanced CT. Enhanced CT shows dilation of the proximal bowel from jejunostomy (arrows: button jejunostomy site)

BOFJ was defined as postoperative bowel obstruction requiring surgery, and caused by the jejunostomy site according to CT or intraoperative findings (Fig. 2).

Diagnostic criteria on CT were proximal small bowel dilation, distal small bowel collapse, or a whirl sign visible on CT at the site of jejunostomy.

The surgical indication for BOFJ was repeated bowel obstruction or peritonitis.

### Statistical analysis

Parametric and non-parametric continuous variables were compared by the t-test and Mann–Whitney test, respectively. Dichotomous variables were compared between groups using the chi-squared test. All statistical analyses were performed using Stata/IC version 16.0 (STATA Statistical Software; Stata Corp., College Station, TX). Two-sided probability (*p*) values of 0.05 were considered significant.

## Results

Six patients (8.5%) were diagnosed with BOFJ (Table 1). For all BOFJ patients, esophagectomy was performed under HALS. Mean duration from esophagectomy to bowel obstruction surgery was 4.0 months (range 0–25 months). Of these 6 patients with BOFJ, all were symptomatic and three had histories of recurrent BOFJ (3, 3, and 5 times).

**Table 1:** The 6 patients with BOFJ after esophagectomy with PFJ

Case	Age range (years) Sex (1 or 2)	Procedure	VD (mm) HD (mm)	Duration (Esophagectomy—BOFJ)	Surgical indication Duration of conservative therapy (days)	Procedure	Operative time (min)	Pattern
1	50–60	HALS	102	6 days	Peritonitis	Adhesiolysis	45	AB
	1		47					
2	80–90	HALS	93	7 days	Peritonitis	Adhesiolysis	34	AB
	1		40					
3	70–80	HALS	120	4 months	Repeated BOFJ (5 times)	Adhesiolysis	56	AB
	1		59		3, 3, 2, 3, 2	Bowel resection		
4	60–70	HALS	95	25 months	Not effective of conservative therapy	Adhesiolysis	89	TI
	1		50		> 7	Bowel resection		
5	50–60	HALS	113	3 months	Repeated BOFJ (3 times)	Adhesiolysis	99	TI
	1		42		5, 3, 2	Bowel resection		
6	60–70	HALS	111	16 months	Repeated BOFJ (3 times)	Adhesiolysis	65	AB
	2		49		2, 2, 3	Bowel resection		

HALS, hand-assisted laparoscopic surgery; VD, vertical distance between navel and jejunostomy; HD, horizontal distance between navel and jejunostomy; BOFJ, bowel obstruction associated with feeding jejunostomy; AB, adhesion + bending; TI, torsion + internal hernia

Laparotomy for bowel obstruction was performed in all cases. Two patients underwent emergency operation due to peritonitis. Four patients underwent one or two attempts at non-operative management with fasting or a long intestinal tube, followed by elective surgery due to repeated BOFJ or the ineffectiveness of conservative therapy (> 7 days). No intestinal necrosis was found in any patients, but 2 patients had bowel torsion, and 4 patients had adhesions and acute bowel bending. No patients had mechanical obstruction due to button jejunostomy.

Two patients were treated using only adhesiolysis at the jejunostomy site, while the remaining 4 patients required resection of the jejunum. Functional end-to-end anastomosis was performed using a linear stapler. Reconstruction of the jejunostomy was needed for 1 patient.

No complications were identified in any of the 6 patients who required surgery.

### Analysis for BOFJ (Table 2)

**Table 2:** Patient characteristics and intraoperative factors in all patients

Variable	Total	With BOFJ	Without BOFJ	*p* value (univariate)
		n (%) or median (range)		
Patients	70	6	64	
*Preoperative factors*				
Age (years)	68 (46–91)	64 (52–82)	69 (46–91)	0.415*
Sex				
Male	65 (93%)	5 (8%)	60 (92%)	0.343^†^
Female	5 (7%)	1 (20%)	4 (80%)	
BMI (kg/m^2^)	19 (12–34)	20 (15–26)	18 (12–34)	0.563**
Serum total protein (mg/dL)	6.9 (5.7–7.9)	7.1 (6.3–7.5)	6.9 (5.7–7.9)	0.297*
Serum albumin (mg/dL)	4 (2.9–4.9)	4.3 (3.3–4.3)	4 (2.9–4.9)	0.735*
Neoadjuvant chemotherapy	14 (20%)	0	14 (22%)	0.200^†^
Adjuvant chemotherapy	50 (71%)	5 (83%)	45 (70%)	0.500^†^
Pathological stage				
I	23 (33%)	4 (17%)	19 (83%)	0.324^†^
II	19 (27%)	1 (5%)	18 (95%)	
III	26 (37%)	1 (4%)	25 (96%)	
IV	2 (3%)	0	2 (100%)	
COPD	4 (6%)	1 (25%)	3 (75%)	0.227^†^
Hypertension	22 (31%)	1 (5%)	21 (95%)	0.415^†^
Current smoker	40 (57%)	5 (13%)	35 (87%)	0.175^†^
*Intraoperative factors*				
HALS	28	6	22	0.002^†^
Distance between navel and jejunostomy				
Straight distance (mm)	66 (37–150)	117 (101–130)	63 (37–150)	< 0.001**
Vertical distance (mm)	54 (17–135)	107 (93–120)	49 (17–135)	< 0.001**
Horizontal distance (mm)	39 (14–60)	48 (40–59)	37 (14–60)	0.020**
Surgical duration (min)	451 (309–613)	409 (347–464)	460 (309–613)	0.015*
Estimated blood loss (mL)	119 (1–1030)	122 (90–150)	114 (1–1030)	0.817**
Length of button (cm)	4.0 (3.0–5.0)	3.5 (3.0–4.0)	4.0 (3.0–5.0)	0.773*

BOFJ, bowel obstruction associated with feeding jejunostomy; BMI, body mass index; COPD, chronic obstructive pulmonary disease; HALS, hand-assisted laparoscopic surgery

^†^Chi-square test

^*^Unpaired t test

^**^Mann–Whitney test

Median duration of postoperative follow-up was 28 months (range 1–78 months). Cancer recurrence was identified in 32 patients.

Comparing patients with and without BOFJ, no significance differences in preoperative factors were identified among patients who underwent esophagectomy. Compared to the without-BOFJ group, the BOFJ group showed a longer straight-line distance between the jejunostomy and navel (117 mm [101–130 mm] vs. 63 mm (37–150 mm), *p* < 0.001) and shorter surgical duration (409 min [347–464 min] vs. 460 min [309–613 min], *p* = 0.015). Furthermore, comparing patients with and without BOFJ, univariate analysis showed a significant difference in HALS (n = 6 vs. n = 22, *p* = 0.002). The laparoscopic group did not include any cases of BOFJ, so we could not perform multivariate analyses between the two groups. Subgroup analysis was performed for the HALS group.

### Subgroup analysis for BOFJ in HALS group (Table 3)

**Table 3:** Characteristics and intraoperative factors in patients who underwent esophagectomy under HALS

Variable	Total	With BOFJ	Without BOFJ	*p* value (univariate)
		n (%) or median (range)		
Patients	28	6	22	
*Preoperative factors*				
Age (years)	67 (52–86)	64 (52–82)	69 (52–86)	0.622*
Sex				
Male	27 (96%)	5 (19%)	22 (81%)	0.051^†^
Female	1 (4%)	1 (100%)	0 (0%)	
BMI (kg/m^2^)	19 (12–26)	20 (15–26)	19 (12–26)	0.462*
Serum total protein (mg/dL)	7.0 (5.9–7.5)	7.1 (6.3–7.5)	6.9 (5.9–7.5)	0.421*
Serum albumin (mg/dL)	4.1 (3.2–4.9)	4.3 (3.3–4.3)	4.0 (3.2–4.9)	0.878*
Neoadjuvant chemotherapy	1 (3.6%)	0	1 (100%)	0.595^†^
Adjuvant chemotherapy	24 (86%)	5 (21%)	19 (79%)	0.851^†^
Pathological stage				
I	9 (32%)	4 (44%)	5 (56%)	0.221^†^
II	6 (21%)	1 (17%)	5 (83%)	
III	12 (43%)	1 (8%)	11 (92%)	
IV	1 (4%)	0	1 (100%)	
COPD	2 (7.1%)	1 (50%)	1 (50%)	0.307^†^
Hypertension	8 (29%)	1 (12%)	7 (88%)	0.466^†^
Current smoker	14 (50%)	5 (36%)	9 (64%)	0.065^†^
*Intraoperative factors*				
Distance between navel and jejunostomy				
Straight distance (mm)	97 (51–150)	117 (101–130)	89 (51–150)	< 0.001*
Vertical distance (mm)	83 (28–135)	107 (93–120)	79 (28–135)	0.010*
Horizontal distance (mm)	47 (22–60)	48 (40–59)	46 (22–60)	0.199*
Surgical duration (min)	417 (309–498)	409 (347–464)	421 (309–498)	0.860*
Estimated blood loss (mL)	145 (5–1030)	122 (90–150)	173 (5–1030)	0.341**
Length of button (cm)	4.0 (3.0–4.0)	3.5 (3.0–4.0)	4.0 (3.0–4.0)	0.475**

BOFJ, bowel obstruction associated with feeding jejunostomy; BMI, body mass index; COPD, chronic obstructive pulmonary disease; HALS, hand-assisted laparoscopic surgery

^†^Chi-square test

^*^Unpaired t test

^**^ Mann–Whitney test

The HALS group included 28 patients.

Univariate analysis revealed a significant difference only in straight-line distance between the jejunostomy and navel as a significant pre- and perioperative factor (117 mm [101–130 mm] vs. 89 mm [51–150 mm], *p* < 0.001). Furthermore, after dividing straight-line distance between the jejunostomy and navel into VD and HD, only VD showed a significant difference (107 mm [93–120 mm] vs. 79 mm [28–135 mm], *p* = 0.010), not HD (48 mm [40–59 mm] vs. 46 mm [22–60 mm], *p* = 0.199). All BOFJ cases showed VD ≥ 90 mm.

### Analysis of HALS and laparoscopic groups (Table 4)

**Table 4:** Comparison of intraoperative factors between HALS and laparoscopic groups

Variable	Total	HALS	Laparoscopic	*p* value univariate
		n (%) or median (range)		
Patients	70	28	42	
*Intraoperative factors*				
Distance between navel and jejunostomy				
Straight distance (mm)	66 (37–150)	97 (51–150)	57 (37–111)	< 0.001**
Vertical distance (mm)	54 (17–135)	83 (28–135)	42 (17–98)	< 0.001**
Horizontal distance (mm)	39 (14–60)	47 (22–60)	32 (14–60)	< 0.001**
Surgical duration (min)	451 (309–613)	417 (309–498)	491 (351–613)	< 0.001*
Estimated blood loss (mL)	119 (1–1030)	145 (5–1030)	75 (1–650)	0.051**
Length of button (cm)	4.0 (3.0–5.0)	4.0 (3.0–4.0)	4.0 (3.0–5.0)	0.030*

HALS, hand-assisted laparoscopic surgery

^*^Unpaired t test

^**^Mann–Whitney test

Univariate analysis showed a significant difference in VD between groups (83 mm [28–135 mm] vs. 42 mm [17–98 mm], *p* < 0.001). These data showed a longer VD in the HALS group than in the laparoscopic group. Other factors including HD, surgical duration, and length of the button also differed significantly between groups (*p* < 0.001, *p* < 0.001, *p* = 0.030).

## Discussion

The incidence of BOFJ after esophagectomy was 8.5% in our study cohort, with all cases arising in the HALS group. Patients with BOFJ showed a significantly longer VD than patients without BOFJ. Our study demonstrated that longer VD between the jejunostomy and navel might represent a risk factor for BOFJ.

Recent clinical guidelines have recommended early postoperative enteral nutrition as a method of reducing major complications, such as infection and anastomotic leakage, postoperative ileus and albumin requirements compared to parenteral nutrition [16, 17]. PFJ is commonly performed during esophagectomy to subsequently maintain adequate enteral nutrition [6–9].

The following are the objectives of PFJ: (1) to prevent villous atrophy and maintain gastrointestinal integrity by promoting peristalsis, blood flow and secretion of digestive juices; (2) to maintain or enhance immune function and reduce operative complications by administration of immunonutrients; (3) to avoid complications of parenteral nutrition such as catheter-associated hematological infection and venous thrombosis, which are related to long-term indwelling central venous catheters; and (4) to avoid physical loss of the mucosal barrier due to long-term disuse of the gut, by promoting bacterial translocation [18].

In a large cohort study of 2495 patients, Lorimer et al. demonstrated that enteral feeding access is associated with improved short-term survival at 90 days and does not prolong the hospital stay [19].

However, PFJ during esophagectomy is associated with a 2–15% incidence of serious complications such as bowel obstruction, intractable jejuno-cutaneous fistula, PFJ-site infection, tube occlusion, and leakage [9–12]. Among these complications, BOFJ is a particularly serious problem because of repeated occurrence and the need for surgical intervention. The incidence of BOFJ has been reported as 0–11.5% in recent reports [10, 12, 18, 20–24]. However, few reports have described risk factors for BOFJ.

Kitagawa [25] mentioned lower adhesion formation after laparoscopic surgery as a potential risk factor for postoperative BOFJ and internal hernia. With gastric mobilization, a large intra-abdominal space is formed on the left side of the jejunostomy, into which the jejunum might invaginate and twist around the feeding jejunostomy. This might explain the higher rate of BOFJ among patients who underwent a treatment with a laparoscopic approach, and shorter distance between the jejunostomy and midline or xiphoid process line in their study group.

Furthermore, Shiraishi et al. [24] suggested that in cases of laparoscopic surgery, the main mechanism of BOFJ is torsion of the mesentery accompanied by migration of the anal-side intestine across the site of stoma fixation to the abdominal wall toward the opposite side, similar to an internal hernia. In their study, closing the space within the triangle formed by the ligament of Treitz, the site of stoma fixation, and the lower pole of the spleen with omentum stuck to the transverse colon (as the so-called curtain method) could prevent BOFJ. Other studies have also reported that prolonged duration of tube feeding or internal hernia space created after the surgery might be risk factors for BOFJ or internal hernia [26, 27].

**Fig. 3:**
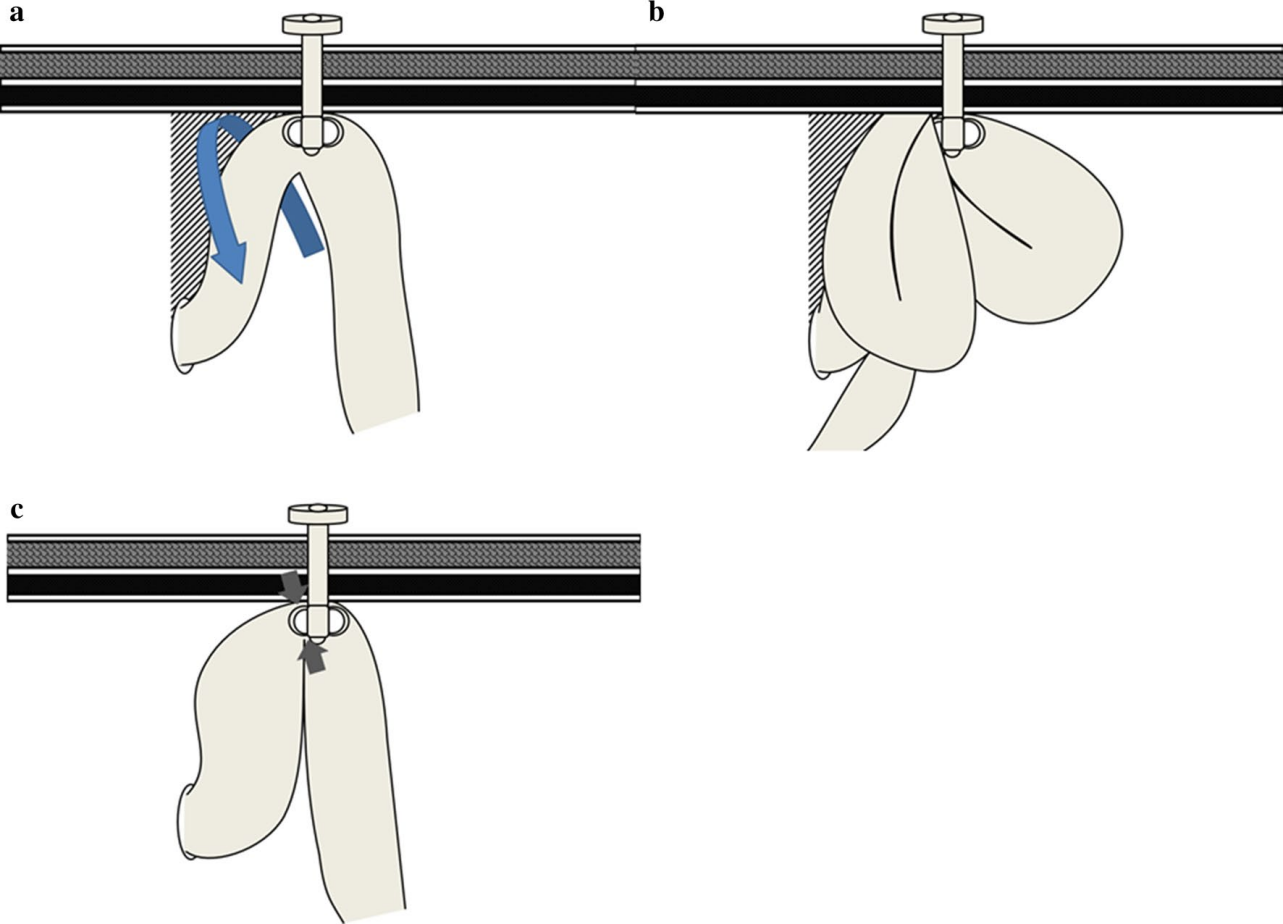
Mechanisms in BOFJ. **a**, **b** Torsion + internal hernia pattern (TI pattern) torsion site (arrow), **c** adhesion + bending pattern (AB pattern). Obstruction site (arrow)

We consider that BOFJ involves several mechanisms. One is the torsion + internal hernia pattern (TI pattern) (Fig. 3a, b) as reported by Shiraishi et al. [24]. The other is the adhesion + bending pattern (AB pattern) (Fig. 3c).

In the TI pattern, the curtain method [24] and fixation at several points or longitudinal fixation to the abdominal wall [28] are feasible procedures to reduce the rate of BOFJ.

However, in our experience, of the 6 patients with BOFJ, only 2 patients showed a TI pattern. Four cases displayed an AB pattern and the main cause of BOFJ was the bending angle between the abdominal wall and jejunostomy. An acute bending angle would cause repeated BOFJ and thus requires reconstruction of jejunostomy. This is why our result for VD from the navel appears very important. No consensus has yet been reached regarding the optimal site of jejunostomy. We consider that when the VD is > 9 cm from the navel, the angle from the ligament of Treitz and jejunostomy becomes steeper. A longer VD might thus be associated with a higher rate of BOFJ.

Furthermore, our button jejunostomy might be associated with a higher rate of BOFJ with the AB pattern. We applied a simple and efficient button jejunostomy that is not prone to dislodgement and employs an easily replaceable feeding button. Button jejunostomy is also relatively comfortable for patients because of the esthetic outcomes and short length [15, 29]. Recent reports [10, 12, 18, 20–24] have applied the conventional Witzel tube jejunostomy. In the Witzel technique, longitudinal sutures are placed on both sides of the feeding tube to imbricate the bowel wall over the feeding tube, creating a serosal tunnel. In button jejunostomy, the fixation area is shorter than that with the Witzel technique, so the AB pattern might result. We consider that PFJ within 9 cm from the navel might be able to prevent BOFJ with the AB pattern.

Recently, some researchers have recommended insertion of the feeding catheter into the duodenum [18] or gastric tube [20, 22], through the round ligament of the liver, rather than through the jejunum to prevent BOFJ.

However, in patients with posterior mediastinal route reconstruction, constructing a feeding gastrostomy with round ligament is sometimes difficult because the location of gastric antrum is separate from the round ligament [22]. Another concern about duodenostomy has been inflammation around the duodenal bulb or pylorus following leakage and/or catheter-related abscess, leading to gastric tube stasis due to edema of the duodenum and/or pylorus [18].

This study showed several limitations, including the fact that it was a retrospective study of data obtained from a single institution with a small number of patients and clear selection bias. Our study included only patients who had undergone button jejunostomy, and whether the same results would be true for conventional Witzel tube jejunostomy remains unclear.

Furthermore, in our study, all BOFJ occurred in the HALS group. Confirmation of whether VD from the navel is truly associated with BOFJ warrants further investigation. We consider HALS as a confounding factor among risk factors for BOFJ. However, our procedure for PFJ was consistent among all patients, so the finding of a significant difference in VD from the navel as a risk factor for BOFJ is crucial.

Studies using data from a large-scale, multicenter registry are necessary to determine risk factors for BOFJ in the future.

## Conclusion

We identified a higher risk of BOFJ among patients under HALS esophagectomy with longer VD between the jejunostomy and navel. Performing PFJ during HALS esophagectomy within 9 cm from the navel might prevent BOFJ.

## Data Availability

The datasets used and/or analyzed during the current study are available from the corresponding author on reasonable request.

## Abbreviations

PFJ: Placement of feeding jejunostomy
BOFJ: Bowel obstruction associated with feeding jejunostomy
HALS: Hand-assisted laparoscopic surgery
VD: Vertical distance
HD: Horizontal distance
CT: Computed tomography
IB: Ideal button
BMI: Body mass index
TI pattern: Torsion + internal hernia pattern
AB pattern: Adhesion + bending pattern
